# Peritumoral abnormalities on dynamic-enhanced CT after brachytherapy for hepatic malignancies: local progression or benign changes?

**DOI:** 10.1007/s00330-022-09074-x

**Published:** 2022-08-18

**Authors:** Guanyu Chen, Dechao Jiao, Sheng Peng, Xi Chen, Yanling Zhang, Letao Lin, Zhihui Zhong, Yong Li, Kaihao Xu, Fujun Zhang

**Affiliations:** 1 Department of Minimally Invasive & Interventional Radiology, State Key Laboratory of Oncology in South China; Collaborative Innovation Center for Cancer Medicine, Sun Yat-sen University Cancer Center, 651 Dongfeng Road East, Guangzhou, Guangdong 510060 People’s Republic of China; 2 Department of Interventional Radiology, The First Affiliated Hospital of Zhengzhou University, Zhengzhou, 450052 People’s Republic of China; 3 Department of Ultrasound, State Key Laboratory of Oncology in South China, Collaborative Innovation Center for Cancer Medicine, Sun Yat-sen University Cancer Center, Guangzhou, 510060 People’s Republic of China; 4 School of Laboratory Medicine and Biotechnology, Southern Medical University, Guangzhou, 510000 People’s Republic of China; 5 Department of Intervention, Zhuhai People’s Hospital, Zhuhai, 519000 People’s Republic of China

**Keywords:** Hepatic malignancies, Brachytherapy, Iodine-125, Radiation injury, CT

## Abstract

**Objectives:**

To determine if dynamic CT can differentiate local progression from radioactive seed-induced peritumoral reaction (RSIPR) after brachytherapy with iodine-125 radioactive seeds (BIRS) for advanced hepatic malignancies.

**Methods:**

Enhanced CT images of seed-implanted lesions between 2006 and 2018 were retrospectively evaluated. Hounsfield units of peritumoral parenchyma were measured and assessed quantitatively. The classification, conversion, consequences, and serological indicators during follow-up were recorded and quantified. Statistical differences were analyzed using a Pearson *χ*^2^ test.

**Results:**

RSIPR was observed in 201 of 290 (69.3%) lesions (161 patients; median age, 55 years; range, 26–79 years), while local progression occurred in 53 lesions. The low density of local progression was much lower than that of RSIPR (*p* < 0.001), and the former did not exhibit iso-/high density in the portal or equilibrium phase. Ring-like enhancement in progressive lesions was also quite different from RSIPR. Local progression rate was lower for lesions with RSIPR than for those without RSIPR (14.9% vs 25.8%; *p* = 0.03), and their doses were different (397.2 Gy vs 120.3 Gy, *p* < 0.001).

**Conclusions:**

Radioactive seed-induced peritumoral reaction has characteristic manifestations on CT images, which is associated with a higher dose of lesions and lower local progression rate. Notably, the enhancement pattern of local progression was distinct from RSIPR and was clearly distinguishable on dynamic-enhanced CT.

**Key Points:**

*• Radioactive seed-induced peritumoral reaction after brachytherapy with* ^*125*^*I seeds for liver malignancies has characteristic manifestations on CT images, which is associated with a higher dose of lesions (397.2 Gy vs 120.3 Gy, p < 0.001), as a focal radiation injury.*

*• Lesions with RSIPR were less likely to develop local progression, while those without RSIPR had a higher rate of local progression (14.9% vs 25.8%; p = 0.03).*

*• The enhancement pattern of local progression after brachytherapy was distinct from radioactive seed-induced peritumoral reaction and was clearly distinguishable on dynamic-enhanced CT.*

**Supplementary Information:**

The online version contains supplementary material available at 10.1007/s00330-022-09074-x.

## Introduction

The most common primary liver malignancy is hepatocellular carcinoma (HCC), which is one of the most common cancers worldwide [1], followed by cholangiocarcinoma. In addition, the liver is one of the most common metastatic organs, including metastases associated with colorectal cancer, melanoma, and pancreatic cancer [2–5].

Brachytherapy with iodine-125 radioactive seeds (BIRS) has promising results for safety and efficacy for the treatment of primary and secondary liver malignancies [6–9]. BIRS enables the release of high-dose radiation into the tumor in a concentrated manner, with a sharp decrease in dose outside the target volume surface, thereby reducing damage to surrounding tissues. As such, BIRS can be performed in places where thermal ablation exposes to complications or to replace external beam radiation therapy (EBRT) when unable to reach the relevant dosage. Accordingly, BIRS offers another treatment option in patients with advanced cancer [10–13].

Based on cost availability, seed imaging capabilities, and dosimetric assessment accuracy, computed tomography (CT) is recommended for routine detection of BIRS [14, 15]. CT is widely used for the detection of local progression. Local progression can be identified as clearly enlarged lesions or new protrusions adjacent to seeds. However, atypical enhancement around some seed-implanted lesions might be confusing, like rim-like arterial enhancement [16, 17], or areas of hypo perfusion, hindering the identification of local progression. Progression may only become undisputable based on delayed temporal changes of enhancement pattern or tumor growth, missing the best time for treatment. To date, there is a paucity of data in the literature on these equivocal abnormal imaging changes. The objective of this retrospective study was to analyze CT findings on peritumoral parenchyma and investigate how local progression could be differentiated from a benign reaction.

## Materials and methods

### Population

Patients who were diagnosed with hepatic malignancies in our center and received BIRS from June 2006 to June 2018 were included in this retrospective study. The treatment process of BIRS is described in *Materials and Methods* in Appendix E1. All patients signed an informed consent form prior to treatment. The Institutional Review Board of Sun Yat-sen University Cancer Center approved this study.

### Inclusion and exclusion criteria

The patient selection was random. Based on the half-life of iodine-125, patients with pre-BIRS CT scans and more than two upper abdominal CT scans at our center within 12 months after BIRS were considered for inclusion. Patients who did not receive any CT scan within 12 months after BIRS, underwent ablation or resection soon after BIRS, and/or lacked normal parenchyma around the lesion were excluded.

### Follow-up and CT scans

Follow-up started at 1 month after BIRS. Multiphase CT scans (i.e., plain, arterial, portal venous, and equilibrium phase) were performed with the following parameters: 120 kVp, 150–300 mA of automatic adjustment, slice thickness of 5 mm and 1 mm, and pitch of 0.984:1. A 2.0 mL/kg non-ionic contrast agent (Omnipaque 300, GE Healthcare) was injected intravenously at a flow rate of 3 mL/s. Upper abdominal scans were performed in the arterial phase (delay of 15–30 s after bolus tracking), portal venous phase (delay of 60–70 s), and equilibrium phase (delay of 180–300 s) after injection. Based on previous reports [18, 19], results of several serological indicators tested concurrently with the CT scan were recorded, including alanine aminotransferase (ALT), aspartate aminotransferase (AST), total bilirubin (TBIL), alkaline phosphatase (ALP), and Child–Pugh classification.

### Imaging evaluation

All images were evaluated at the liver window (WL: 93, WW: 109) by three readers, including two senior radiologists with more than 15 years of experience (F.Z. and D.J.) and one radiology resident with 2.5 years of experience (G.C.). CT attenuation values were measured with the tools of Picture Archiving and Communication System (Centricity RIS, GE Healthcare) as tissue density. When measuring CT attenuation values in different months or phases, an effort was made to select the same area at the same level to determine the average. Considering that small differences in contrast agent administration and image acquisition time can affect the results, a difference of 10 HU and above was defined as a significant difference in tissue density based on previous literature [20], i.e., |ΔCT|≥ 10 HU. ΔCT was defined as the CT attenuation value of the peritumoral abnormalities minus that of the surrounding normal liver tissue, or the difference of those in different phases, i.e., ΔCT_P-A_ referred to the CT attenuation value of the portal phase minus the arterial phase, and ΔCT_V-P_ was that of the equilibrium phase minus the portal phase. When its value was positive, it meant that the peritumoral abnormality was high density, i.e., ΔCT_high_ and the negative value was low density, i.e., ΔCT_low_. These ΔCTs were all calculated. The interval between BIRS and abnormal CT findings was calculated, and the duration of abnormal CT findings was measured. The classification, conversion, and consequences of peritumoral abnormalities were quantified.

### Threshold dose and relative diameter

Similar to matched peripheral dose, threshold dose was defined as peripheral exposure dose of visible peritumoral abnormalities and was measured using D90 (the exposure dose of 90% volume of tumor). Cases with complete abnormalities were selected, and those with minimal peritumoral tissue were excluded. CT images of the maximum abnormalities (typically at 1–3 months after BIRS) were transmitted to the treatment planning system. Abnormal areas were delineated at the liver window, and the volume and threshold dose were calculated by a physicist with 5 years of experience (Z.Z.). For greater comparability, relative diameters were calculated using the size of lesions/seed areas as a reference. Artifacts from metal seeds occasionally interfered with our judgment of the lesion boundary, and lesions occasionally contracted significantly or even resolved completely. A seed area delineated at the bone window (WL: 350, WW: 1500) subsequently replaced the lesion area. The maximum diameters were measured, and the relative diameters were calculated using the following formula.

$$ {D}_{rel}=\frac{D_{abn}}{D_{l/s}} $$

*D*_*rel*_ refers to the relative diameter, and *D*_*abn*_ and *D*_*l*/*s*_ refer to the maximum diameters of abnormalities and lesions/seed areas, respectively.

### Statistical analysis

The sample size was estimated using PASS 15.0 (NCSS Statistical software) with the following parameters: power of .90, alpha of .05. The hypothetical proportions of lesions with or without peritumoral abnormalities when the doses are greater than 120Gy were .75 and .05, respectively. Statistical analyses were performed using SPSS 26.0 (IBM) or GraphPad Prism 8 (GraphPad Software). The significance level was set to .05. The normality test was used to assess the normality of data and no data points were removed from the analysis. Pearson’s *χ*^2^ or Fisher’s exact test was used to compare categorical data. An independent *t*-test was used to evaluate the significance of the difference in D90 between lesions with or without peritumoral abnormalities.

## Results

### Patient characteristics

**Fig. 1:**
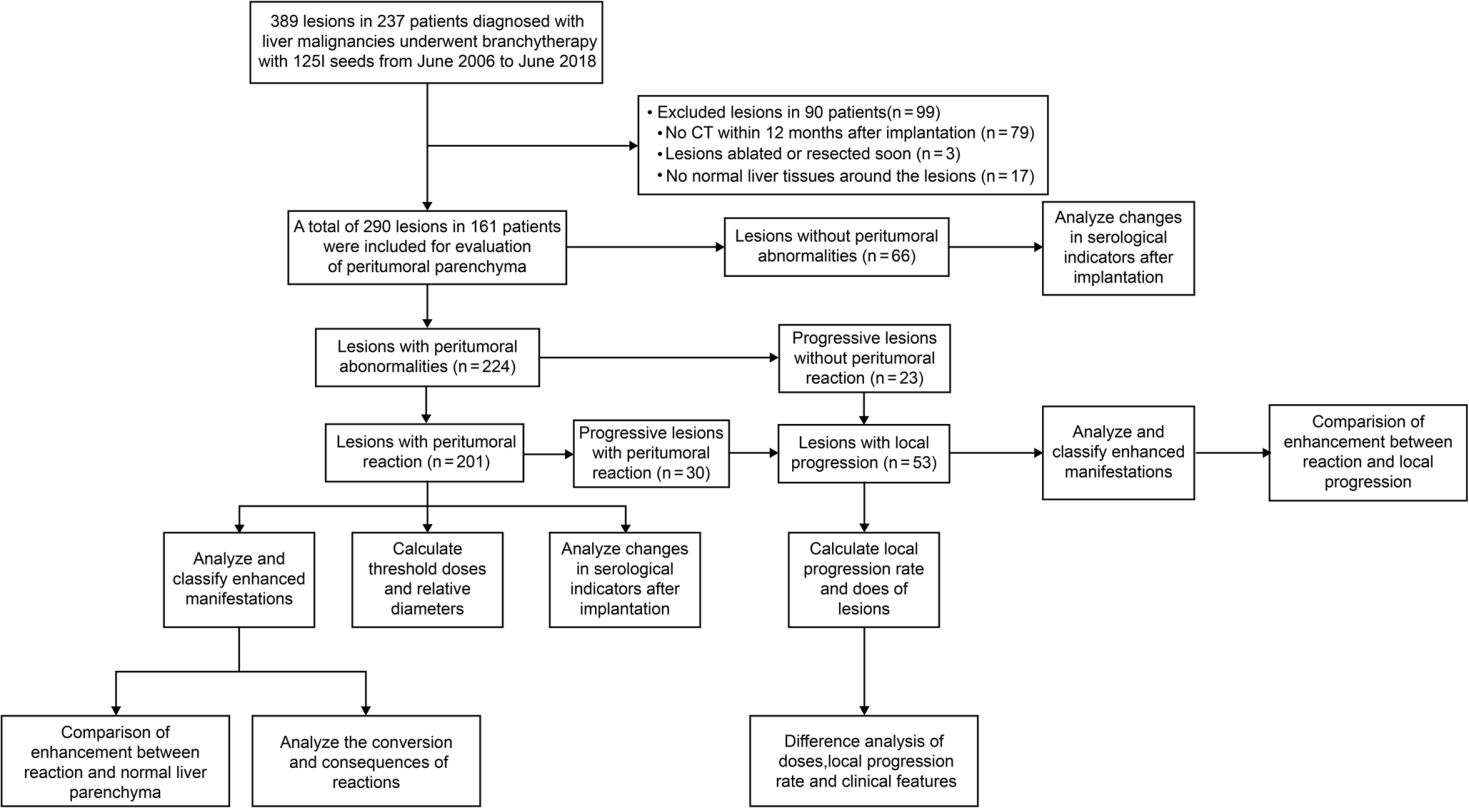
Flowchart depicting an overview of the study and the selection and analysis of cases

In total, 161 patients with 290 lesions were enrolled, including 100 men and 61 women (Table 1). Of the 237 recruited patients, 99 lesions in 90 patients were excluded based on follow-up profiles (Fig. 1). Forty-two patients had new seed-implanted lesions during follow-up, and 16 patients with multiple lesions had no abnormalities in all lesions. The median age was 55 years (range, 26–79 years). In total, 1611 CT scans were reviewed. The median number of post-BIRS CT scans for each lesion was 4 (range, 1–20). The median follow-up time was 9 months (range, 1–68 months) until the end of follow-up for all lesions in June 2020.

**Table 1:** Demographic and clinical characteristics of patients/lesions

Characteristic	No. of patients/lesions
Total patients (lesions), *n*	161 (290)
Sex, patients, *n*	
Male (%)	100 (62.1)
Female (%)	61 (37.9)
Age, patients, *n*	
Median (range)	55 (26**–**79)
< 35 (%)	7 (4.3)
35–60 (%)	104 (64.6)
≥ 60 (%)	50 (31.1)
Organization sources, lesions, *n*	
Primary (%)	126 (43.5)
Secondary (%)	164 (56.5)
Histopathological types, lesions, *n*	
Hepatocellular carcinoma (%)	96 (33.1)
Cholangiocarcinoma (%)	28 (9.7)
Melanoma (%)	30 (10.3)
Nasopharyngeal carcinoma (%)	28 (9.7)
Gastrointestinal malignancies (%)	72 (24.8)
Gynecological malignancies (%)	16 (5.5)
Others (%): pancreatic, kidney, lung, gallbladder, etc.	20 (6.9)
Child–Pugh classification before BIRS, lesions, n*	
A (%)	281 (97.9)
B (%)	6 (2.1)
C (%)	0 (0.0)
Intrahepatic treatment before BIRS, lesions, *n*	
MWA/RFA	131
TACE	76
HAIC/chemotherapy	61
Surgical resection	40
Others: PEI, CIK, radiotherapy, Solafini, etc.	43
None	40
Side effects after BIRS, no. of operations, *n* (*n* = 264)	
Pain (%)	18 (6.8)
Others (%): fever, pneumothorax, biliary infection, etc.	14 (5.3)
None (%)	232 (87.9)

Abbreviations: *BIRS*, brachytherapy with iodine-125 radioactive seeds; *MWA*, microwave ablation; *RFA*, radiofrequency ablation; *TACE*, transcatheter arterial chemoembolization; *HAIC*, hepatic arterial infusion chemotherapy; *PEI*, percutaneous ethanol injection; *CIK*, cytokine-induced killer

*There were three cases with missing data

### Peritumoral parenchymal abnormalities on enhanced CT

**Fig. 2:**
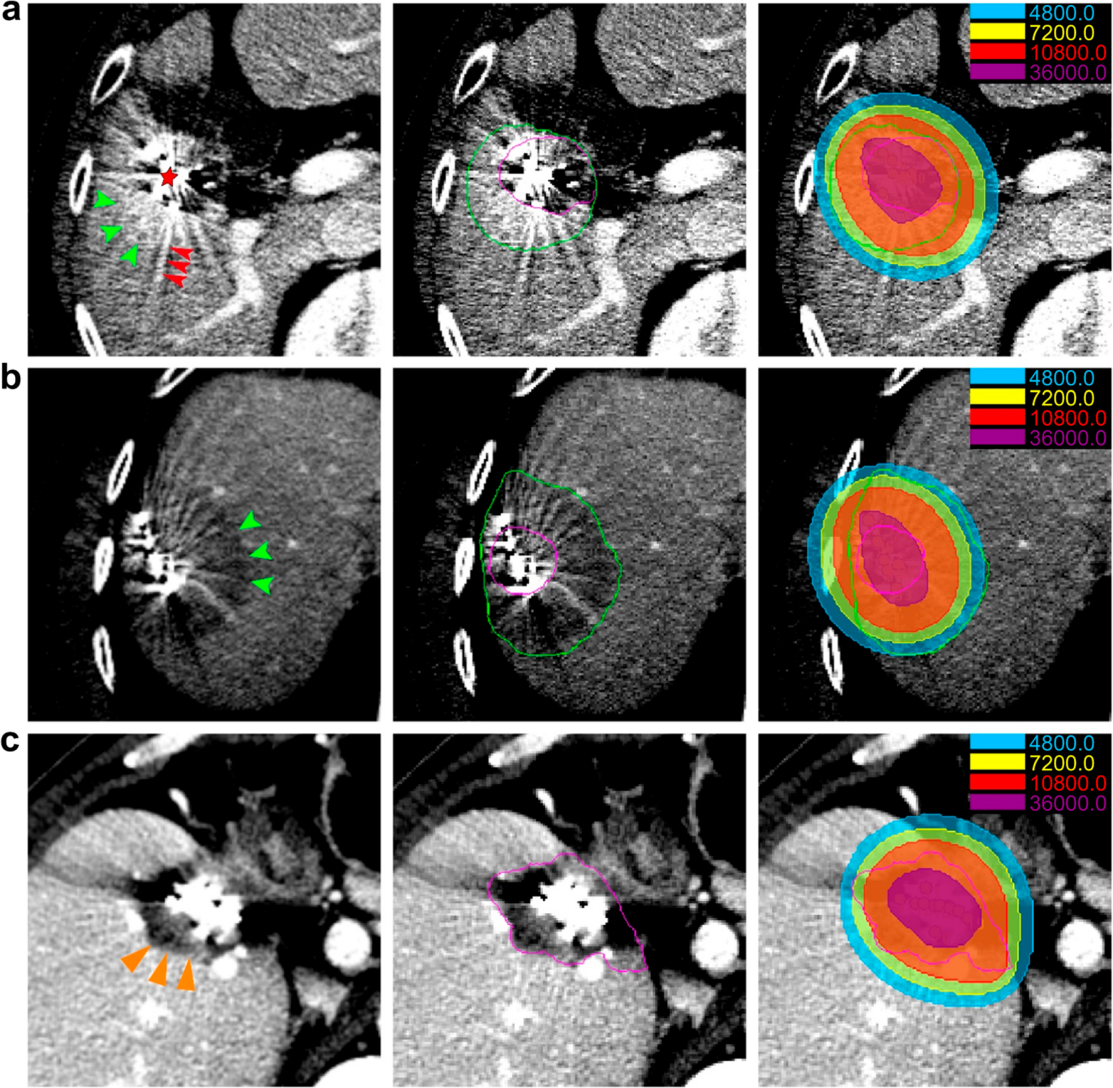
Peritumoral parenchymal abnormalities after brachytherapy on enhanced CT. A high- (**a**) and low-density (**b**) peritumoral reaction (green arrows) and normal peritumoral parenchyma (**c**) on enhanced CT are shown (left). The red star (**a**) marks the seeds, the red arrow refers to artifacts, and the orange arrow (**c**) refers to the progressive lesion. The lesion (purple) and reaction area (green) were delineated (middle). The isodose lines were calculated and drawn, which approximated the shape of the reaction (right). The doses represented by different colors are shown. Unit: cGy

Abnormal peritumoral enhancement was observed in 224 of 290 lesions (77.2%), manifesting as high/low density in the same phase, compared to the surrounding liver (Fig. 2). Peritumoral abnormalities in 201 lesions were identified as benign changes in subsequent scans and were considered to be radioactive seed-induced peritumoral reaction (RSIPR); whereas 53 lesions were identified as local progression, 30 of which initially appeared as benign RSIPR, then developed into progressive lesions later.

### Enhancement pattern of local progression

**Fig. 3:**
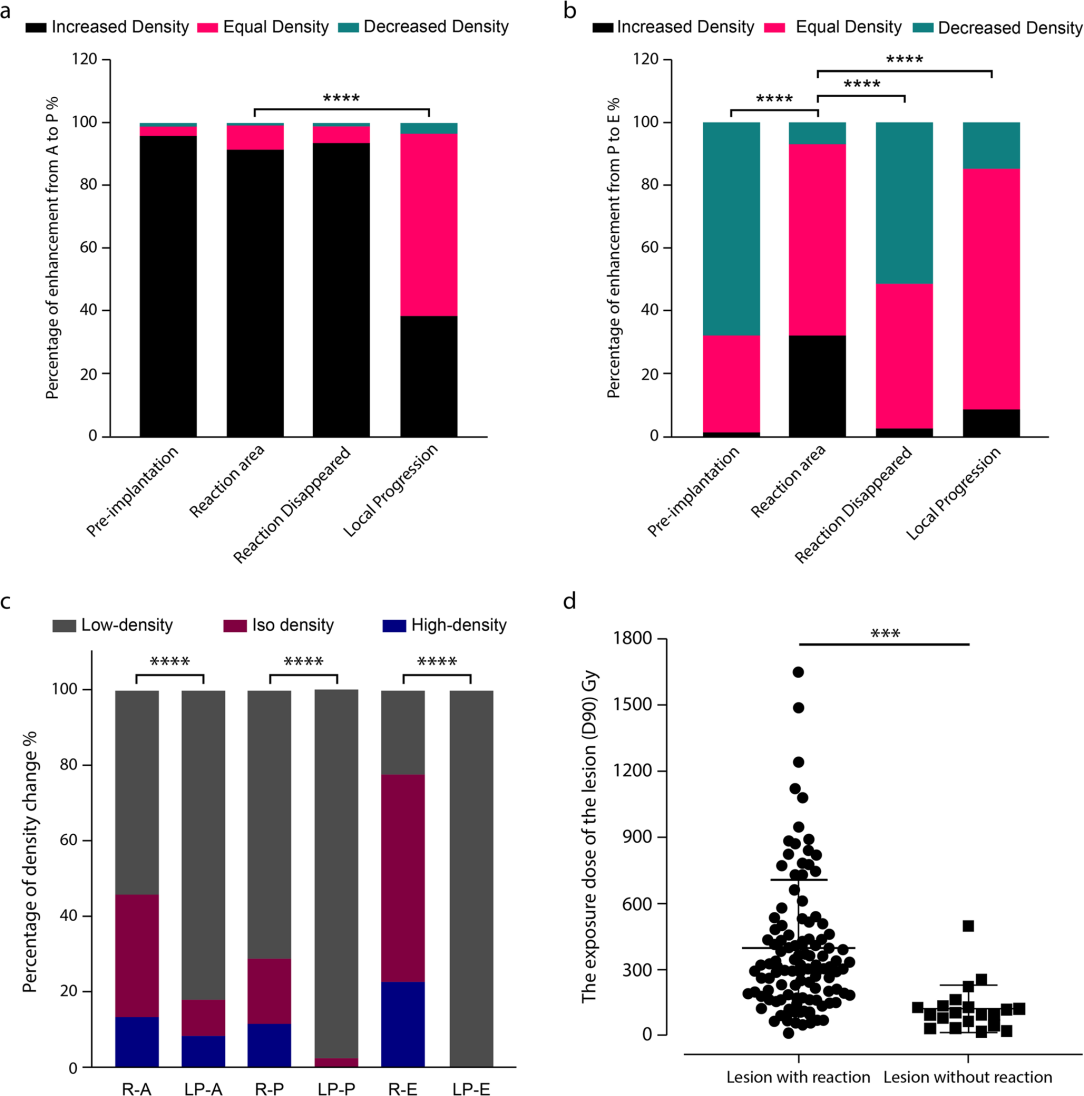
Comparison of enhancement pattern and dose. Comparison of self-enhancement from A to P (**a**) and from P to V (**b**) between pre-implantation, reaction area, disappearance of reaction, and local progression. **c** Comparison of enhancement pattern between reaction and local progression. **d** Comparison of D90 between lesions with and without reaction (*p* = 0.0001). R, reaction; LP, local progression; A, arterial phase; P, portal venous phase, E, equilibrium phase (***p < 0.001, ****p < 0.0001)

All the progressive cases had been characterized. The density of 58.3% of them showed no significant change from the arterial to portal phase, and 77.1% of them from the portal to equilibrium phase, implying a lack of perfusion (Fig. 3a–b). Of them, 38.1% enhanced from the arterial to portal phase, while 14.5% attenuated from the portal to equilibrium phase. Here, central densities were compared, and high-density rings were not quantified.

**Fig. 4:**
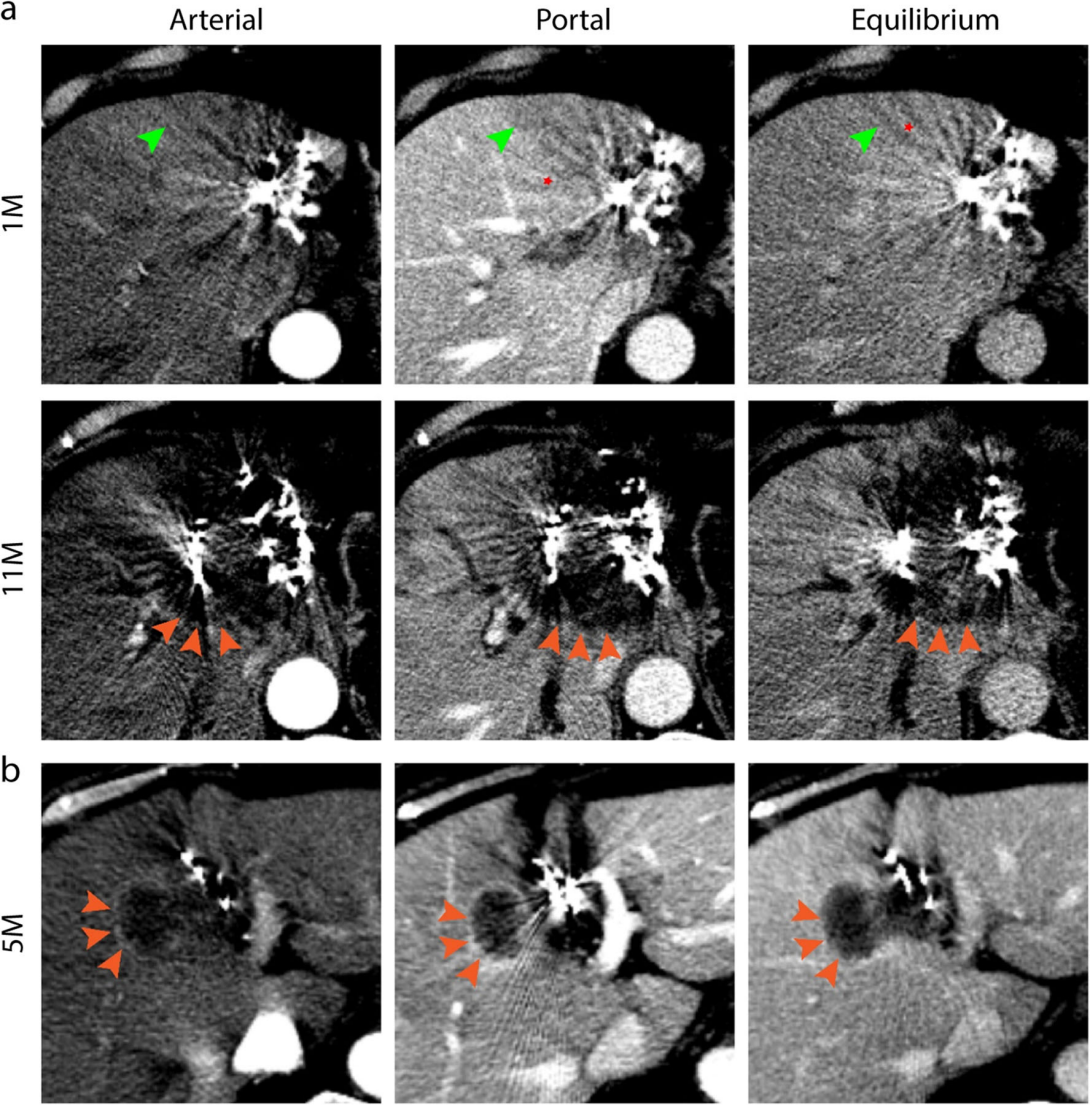
Manifestations of local progression. **a** Recurrence after resection in a 73-year-old woman with cholangiocarcinoma that was seed-implanted, showing a type I reaction 1 month later (green arrows) with several artifacts (red stars). Even after the second implantation, the lesion was still enlarged (orange arrows) at the final follow-up (11 months after the first), exhibiting a single low density in three phases. **b** A new elliptical protrusion (orange arrows) appeared beside the seeds at 5 months in a 78-year-old woman with rectal cancer, exhibiting a high-density ring surrounding a low density

Quantification of density changes in progressive lesions was performed (Table 2). Overall, 65.9% of progressive lesions exhibited low density, whereas only 19.2% and 14.9% appeared as high and iso-density, respectively. The mean of ΔCT_high_ was 19.0 ± 7.0 HU, showing no difference from that of RSIPR (*p* = 0.69); while the mean of ΔCT_low_ was −33.7 ± 16.2 HU, which was significantly lower than that of RSIPR (*p* < 0.001). Progressive lesions usually exhibited two manifestations: a single density, meaning only high density or low density (49.0%, Fig. 4a), and a low density surrounded by a high-density ring (51.0%, Fig. 4b). The latter often appeared as a rim-like arterial enhancement. Only 8.3% of progressive lesions exhibited a single high density in the arterial phase, all of which were HCC, while 82.1% of those appeared as low density (Fig. 3c). Almost all progressive lesions showed hypodensity in the portal (97.6%) and equilibrium phase (100%), similar to arterial enhancement with washout in subsequent phases.

**Table 2:** Quantification of enhanced density in progressive lesions*

Manifestations	Arterial phase/HU		Portal phase/HU		Equilibrium phase/HU	
	High	Low	High	Low	High	Low
Mean ± SD	+21.3 ± 7.3	−23.7 ± 10.8	+16.7 ± 6.5	−43.1 ± 16.8	+15.4 ± 3.9	−32.5 ± 14.1
Max/min	+39.0	−55.0	+31.0	−87.0	+27.0	−68.0
Median	+21.0	−23.0	+14.5	−40.0	+15.0	−29.0

*A difference of 10 HU and above was defined as a significant difference in tissue density, that is, |ΔCT|≥ 10 HU, using the adjacent normal tissue as a reference. *HU*, Hounsfield unit

The median interval to local progression was 11 months (range, 2−31 months), with a median interval of 14 months (range, 4−27 months) for HCC, while more than 98% of radionuclides had decayed at this time, so that the local control had been significantly diminished.

### RSIPR characteristics and classification

RSIPR enhanced significantly from the arterial to portal phase, similar to normal liver tissue, whereby ΔCT_P-A_≥ +10 HU (enhancement of normal peritumoral parenchyma before BIRS and the RSIPR, 159 of 166 vs. 537 of 588, respectively; *p* = 0.09, Fig. 3a). Normally, an attenuation or maintenance of a similar density occurs in the equilibrium phase, i.e., ΔCT_V-P_≤ − 10 HU (68.0%) or |ΔCT_V-P_|<10 HU (30.7%). Of RSIPRs, 60.8% exhibited |ΔCT_V-P_|< 10 HU, and 32.2% exhibited ΔCT_V-P_≥ +10 HU (*p* < 0.001), indicating further enhancement (Fig. 3b).

**Fig. 5:**
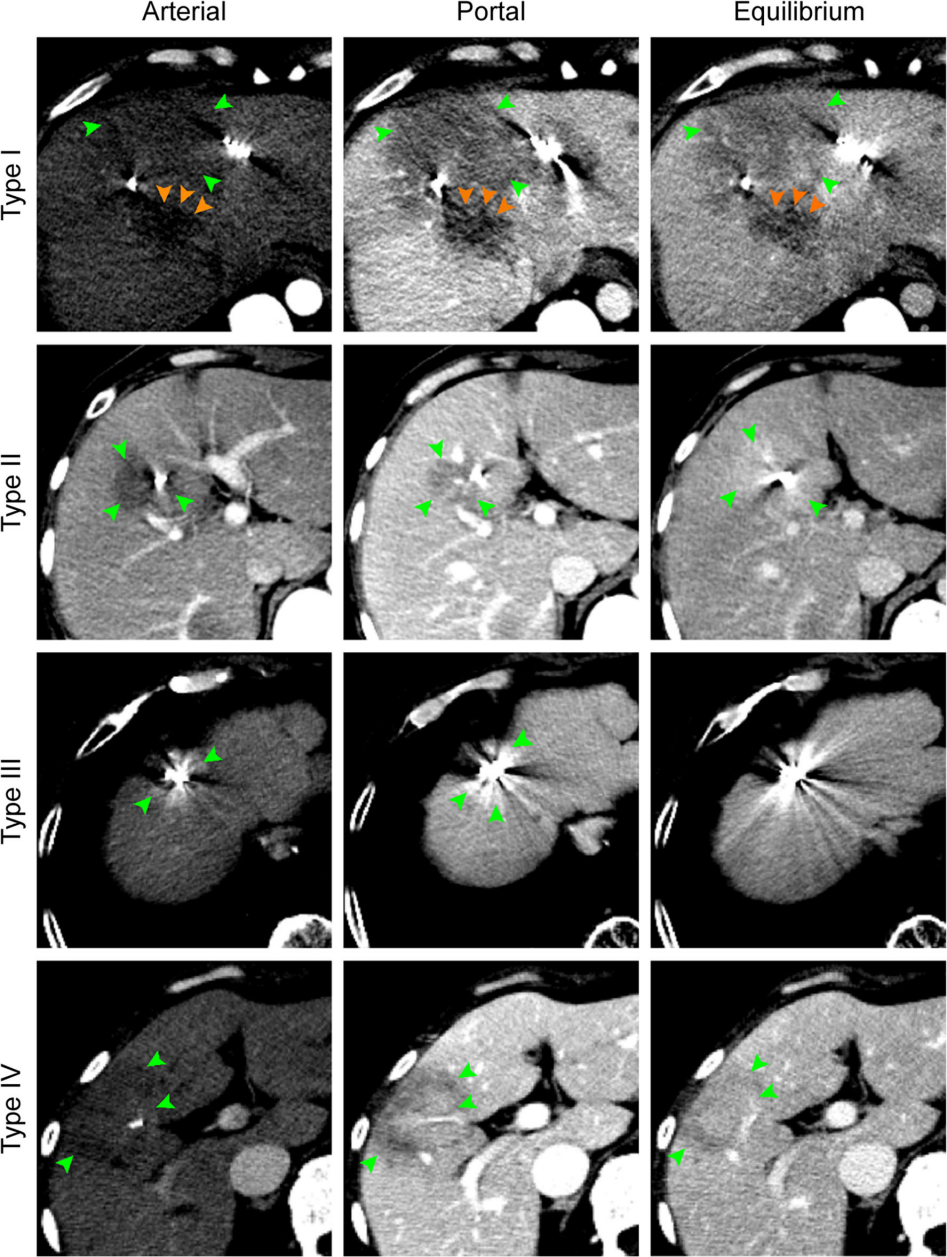
Four types of radioactive seed-induced peritumoral reactions (green arrows). Type I: a 50-year-old man with rectal cancer was seed-implanted in three intrahepatic metastases simultaneously, appearing as a large area of low-low-low 1 month later. Compared to that in previous phases, the density in the equilibrium phase increased. An enlarged adjacent metastasis (orange arrows) had a lower density in all phases. Type II: a 41-year-old woman with an intrahepatic metastasis from cervical cancer was seeded due to its proximity to the gall bladder. To avoid artifacts, the upper images adjacent to seeds were selected, appearing as low-low-high 1 month later. Type III: a 72-year-old man with hepatocellular carcinoma had been implanted for 16 months, showing a high-high-high density. Type IV: a 47-year-old woman with melanoma. The reaction presented as a low-density band surrounding an iso-density at 5 months post-implantation. The low density became clearer in the portal phase, but the area contracted, while the iso-density area expanded. The enhancement was heterogeneous

**Fig. 6:**
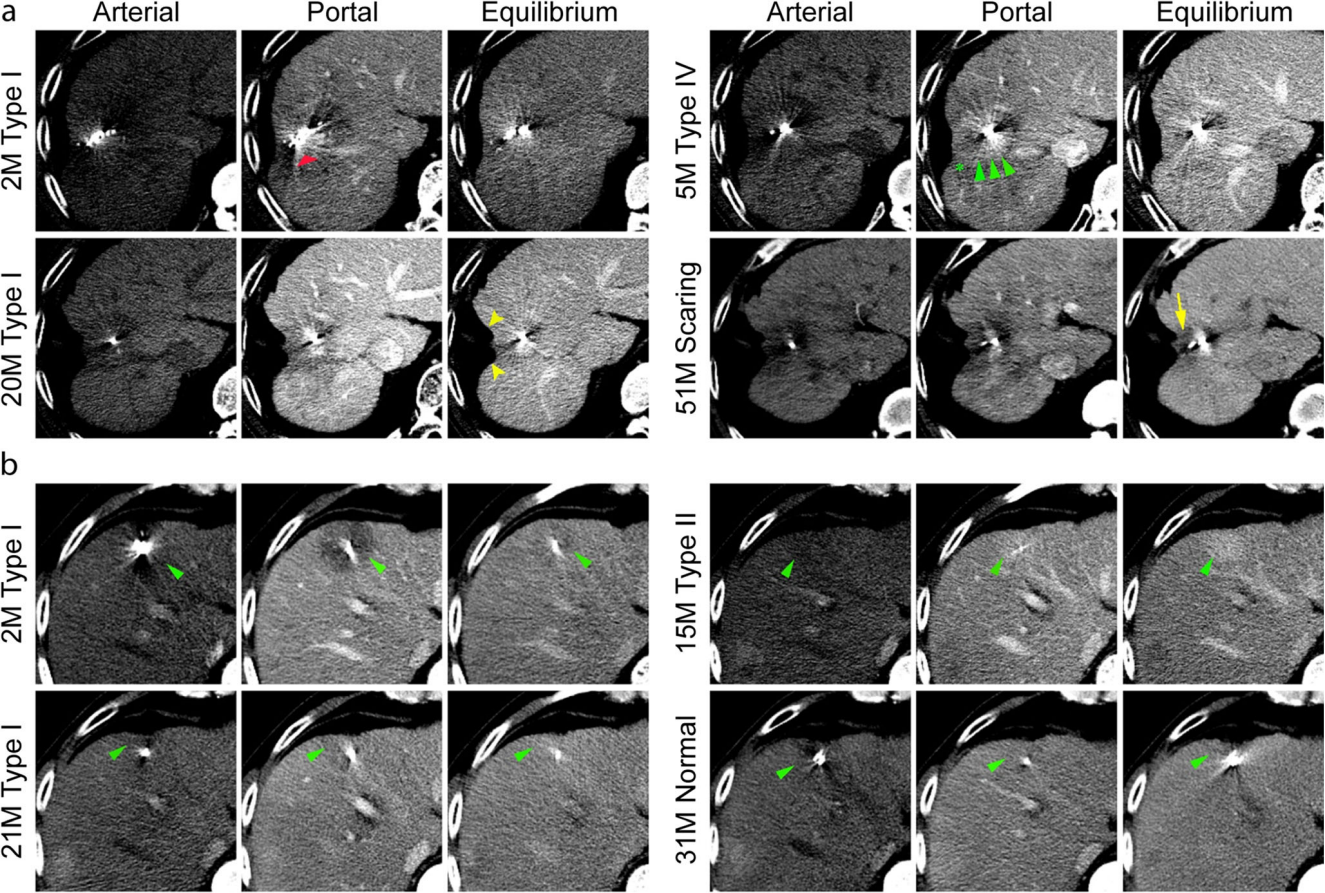
Typical conversion and consequence. **a** The initial reaction was type I with several artifacts (red arrow) in a 54-year-old man with hepatocellular carcinoma. The reaction converted to type IV at 5 months with a low-density band (green star) surrounding a high density (green arrow). At 20 months, the reaction appeared as type I again, with local atrophy (yellow arrow). At 51 months, scarring around the seeds was observed, showing a low density (yellow arrow). **b** CT images at 2, 15, and 21 months post-implantation demonstrated a mutual conversion between types I and II (green arrow) in a 58-year-old man with hepatocellular carcinoma. The reaction recovered to normal at 31 months

The density differences of RSIPR in different phases were quantified (Table 3). In general, the mean of ΔCT_high_ and ΔCT_low_ was 18.6 ± 7.7 HU and −20.2 ± 8.9 HU, respectively. However, the manifestations of RSIPR in triphasic enhancement were more complex than that of local progression. Based on the combination of manifestations in different phases, RSIPR could be classified into four types (Table 4 and Fig. 5). Type I exhibits iso-/low density in all phases, and type II exhibits arterial iso-/low density, but high/iso-density in subsequent phases, while type III exhibits arterial high density. Type IV is characterized by an iso-/low density surrounding a high/iso-density in at least one phase. Low-low-iso was the most common type (25.7%), followed by iso-low-iso (16.7%) and low-low-low (15.5%), highlighting type I as the most common type. But the types were not static (Fig. 6). Notably, the conversions of different types exhibited characteristic patterns (Table E1). The most common types of conversions were types II, III, and IV (Table E2), and type I was the most common type before returning to normal (66.7%). Of 201 cases, 82 (40.8%) finally recovered to normal, and 26 (12.9%) evolved into atrophy or scarring (Table E1 and Fig. 6). Of note, 14.9% (30 of 201) of lesions with RSIPR exhibited local progression, compared with 25.8% (23 of 89) of lesions without RSIPR (*p* = 0.03) .

**Table 3:** Quantification of enhanced density in peritumoral reaction areas*

Manifestations	Arterial phase/HU (*n* = 637)		Portal phase/HU (*n* = 666)		Equilibrium phase/HU (*n* = 575)	
	High	Low	High	Low	High	Low
Mean ± SD	+21.5 ± 10.0	−16.5 ± 5.5	+17.8 ± 6.4	−24.0 ± 10.0	+17.3 ± 5.9	−16.5 ± 5.8
Max/min	+64.0	−41.0	+38.0	−58.0	+36.0	−36.0
Median	+18.0	−15.0	+16.0	−22.0	+16.0	−15.0
No. of CT images (%)	85 (13.3)	345 (54.2)	77 (11.6)	475 (71.3)	130 (22.6)	128 (22.3)

*A difference of 10 HU and above was defined as a significant difference in tissue density, that is, |ΔCT|≥ 10 HU, using the adjacent normal tissue as a reference. HU, Hounsfield unit

**Table 4:** Classification and characteristics of radioactive seed-induced peritumoral reaction

Classification	Arterial phase	Portal phase	Equilibrium phase	No. of CT scans (*n* = 556) (%)*	No. of lesions with initial reaction (*n* = 199) (%) **
Type I	Iso/low	Iso/low	Iso/low	365 (65.6%)	139 (69.8%)
Type II	Iso/low	High/iso/low	High/iso	61 (11.0%)	19 (9.6%)
Type III	High	High/iso/low	High/iso	50 (9.0%)	17 (8.5%)
Type IV	Two density changes occurring simultaneously in at least one phase: low and high, low and iso, iso and high.			80 (14.4%)	24 (12.1%)

*There were 32 scans that could not be classified as type I or type II due to the lack of venous phase

**There were 2 lesions that could not be classified as type I or II due to the lack of venous phase

The median interval of RSIPR was 2 months (range, 1−8 months), and the shortest median duration was 4 months (range, 1−60 months). High-density RSIPR often disappeared within 12−16 months, while low-density RSIPR persisted for longer, even until the end of follow-up (60 months).

### Factors related to RSIPR

Changes in ALT, AST, ALP, TBIL, and Child–Pugh classification were quantified (Table E3). Only ALP exhibited different values between pre- and post-BIRS, regardless of RSIPR (63 of 199 vs. 249 of 507, *p* < 0.001; 34 of 89 vs. 199 of 365, *p* = 0.01). The difference in ALP ceased with the disappearance of RSIPR (63 of 199 vs. 90 of 302, *p* = 0.66).

Multiple single-variable analyses were performed to identify the factors underscoring these reactions in seed-implanted cases (Table E4). RSIPR exhibited significant differences between sexes, organization sources, histopathological types, and lesion positions (see Appendix E1 for details). The averaged D90 was greater for lesions with RSIPR than for those without RSIPR (397.2 ± 307.3 Gy vs 120.3 ± 105.3 Gy, *p* < 0.001, Fig. 3d). Dosimetric calculation revealed that RSIPR shape approximated the isodose line (Fig. 2). The averaged D90 of RSIPR was 66.7 ± 41.7 Gy (range, 11.8–265.2 Gy), median volume was 42.6cc (range, 2.0–440.6 cc), and averaged relative diameter was 2.89 ± 0.88 (range 1.12–5.87).

### Differentiation of RSIPR and local progression

During follow-up, we identified several cases that were actually misevaluations of RSIPR. We therefore compared the enhancement patterns of RSIPR and progressive lesions after characterizing them separately (Table 5). Of 58 progressive lesions from non-HCC, 26 (44.8%) exhibited a single triphasic low density, while their densities were lower than those of RSIPR and distinct from type I (Fig. 5). All progressive lesions exhibited low densities in the equilibrium phase, while type II appeared as high/iso-density distinctly. Progressive lesions from HCC exhibited a single arterial high density, but they washed out in subsequent phases, which was distinct from type III. Type IV always appeared as wider bands, and different ΔCT between phases, even reverting to one density in subsequent phases, and a high-density ring surrounding a low density was not observed in RSIPR (Fig. 5). In contrast, those progressive lesions with ring-like arterial enhancement never exhibited a low density surrounding a high density. And 92% of the rings persisted in all three phases. Besides, the median time to local progression was later than that of RSIPR (11 months vs. 2 months, *p* < 0.001), and vessels in the RSIPR area were observed without invasion (Fig. E1).

**Table 5:** Differentiation of radioactive seed-induced peritumoral reaction (RSIPR) and local progression

Phases	RSIPR		Local progression	
Arterial phase	Low	Type I/II/IV	Low	ΔCT is smaller than that of RSIPR
	Iso	Type I/II/IV	Iso	
	High	Type III/IV	High	Only HCC
	Iso-/low density surrounding high/iso-density	Type IV	High density surrounding low/iso-density	
Portal phase	Low	Type I/II/III	Low	ΔCT is smaller than that of arterial phase
	Iso	Type I/II/III/IV	Iso	
	High	Type II/III/IV	-	
	Iso-/low density surrounding high/iso-density	Type IV	High/iso-density surrounding low density	
Equilibrium phase	Low	Type I	Low	
	Iso	Type I/II/III/IV	-	
	High	Type II/III/IV	-	
	Iso-/low density surrounding high/iso-density	Type IV	High/iso-density surrounding low density	

## Discussion

Due to its safety and efficacy, brachytherapy with ^125^I radioactive seeds (BIRS) has emerged as a treatment option for patients with advanced cancer. Evaluation of peritumoral parenchyma post-BIRS is necessary to distinguish local progression. Using quantitative analysis of enhanced CT images, we observed that the enhancement pattern of radioactive seed-induced peritumoral reaction (RSIPR) was quite different from that of local progression (*p* < 0.001). Notably, the local progression rate was lower for lesions with than for those without RSIPR (*p* = 0.03), while the dose of the former was greater than that of the latter (*p* < 0.001). We propose that RSIPR is a benign focal liver injury which is clearly distinguishable from local progression.

External beam radiation therapy (EBRT) for HCC is prone to be complicated by radiation-induced liver disease (RILD) due to the low tolerance of the liver. Prior studies have shown that RILD refers to severe hepatotoxicity caused by high-dose radiation [18–22]. The irradiated liver undergoes a series of pathological changes, typically manifesting as veno-occlusive disease, and corresponding abnormalities are observable in CT images [18, 22]. Initially, a low density appears on CT (type I) due to endothelial edema, venous outflow obstruction, and liver lobule congestion. Occluded outflow decreases portal-vein inflow, resulting in increased compensatory arterial inflow and high density in the arterial phase (type III). Due to persistent fibrotic deposits, the outflow tract remains blocked, such that the contrast agent is retained, resulting in a high density in the post-enhancement phase (type II). These pathological changes are heterogeneous, which may underscore type IV. Liver lobules may collapse or scar, resulting in a low density (type I), or may regenerate, rendering these changes recoverable. RILD usually manifests as typical clinical symptoms and elevations in multiple serological indicators [23, 24]. In this study, BIRS did not cause obvious side effects during follow-up, and ALP was the only indicator that differed significantly between pre- and post-BIRS, which suggests that ALP is the most sensitive serum marker of RILD [18, 19]. Focal liver injury, referring to focal radiation-induced liver changes revealed by imaging [18], is proposed as a factor underpinning RSIPR.

To date, there have been no reports on RSIPR imaging. However, RILD has been evaluated using CT imaging and can be classified into three types [23, 25]. The Chiou types did not consider the equilibrium phase, while the two Kimura types were consistent with types I and III reported herein. Each type in this study comprised a combination of multiple triple-phase appearances. The portal low densities and equilibrium high densities, but not arterial, have been quantitatively evaluated [20]. In contrast, the high/low densities observed in this study could occur in any phase. Conversion of types has also been reported [20, 23]. Notably, we observed that type I most commonly appeared at the beginning and end, and the other types were predominantly transition types, corresponding to pathology.

A prospective study used a threshold dose of loss of function to describe the irradiated liver volume [26], while we measured a threshold dose of RSIPR, with an average of 66.7 ± 41.7Gy as the total cumulative dose. For a more intuitive measurement, an averaged relative diameter was calculated, indicating that the diameter of RSIPR was 2.89 ± 0.88 times that of lesions.

All of the above is to characterize RSIPR for easy discrimination from local progression. Tumor enlargement and early arterial enhancement are key factors of differentiation for recurrent HCC [27]. In our study, the enhancement pattern of progressive lesions is simpler than that of RSIPR. Similar to original intrahepatic malignancies, advanced HCC exhibits characteristic arterial enhancement with washout in subsequent phases [16, 17], and other intrahepatic malignancies manifest as triphasic hypodense with poor perfusion, or ring-like enhancement, which was clearly distinct from RSIPR. Moreover, the equilibrium phase was important for differentiation.

To our knowledge, there is no report focusing on the differentiation of RSIPR from local progression after BIRS. Yet still some limitations of our study should be noted. Seed artifacts were the main factor interfering with the evaluation, which were typically radial, with alternating high and low densities. RSIPR appeared as an abnormal band surrounding the lesion, perpendicular to the direction of artifacts. In this regard, spectral CT imaging has been reported to substantially reduce artifacts, improving the visualization of seeds and surrounding areas [28]. Besides, since many advanced patients did not receive CT scans after BIRS, cases without RSIPR and follow-up time are insufficient. Regarding the calculation of dose and local progression rate, the selectable cases are also insufficient, regardless of RSIPR. Prospective studies are warranted to clarify the relationship between these parameters.

In summary, we characterized radioactive seed-induced peritumoral reaction, which was associated with a higher dose and lower local progression rate. Furthermore, its enhancement pattern was distinct from that of local progression, which may facilitate the distinction of the latter on enhanced CT.

## Supplementary information

ESM 1 (DOCX 306 kb)

## Abbreviations

ALP: Alkaline phosphatase
BIRS: Brachytherapy with iodine-125 radioactive seeds
D90: Exposure dose of 90% volume of tumor
EBRT: External beam radiation therapy
RILD: Radiation-induced liver disease
RSIPR: Radioactive seed-induced peritumoral reaction
