# Supplementary material for: Peritumoral abnormalities on dynamic-enhanced CT after brachytherapy for hepatic malignancies: local progression or benign changes?
Source: Eur Radiol. 2022 Aug 18;32(10):7307–19. doi: 10.1007/s00330-022-09074-x (PMC9474341; doi:10.1007/s00330-022-09074-x)
Supplement: Supplementary file 1 — (DOCX 306 kb) [file 330_2022_9074_MOESM1_ESM.docx]

**Supplemental Materials**

**Appendix E1**

**Methods and Materials**

**125I brachytherapy**

Most patients receiving brachytherapy had advanced or unresectable tumors or poorly controlled intrahepatic lesions during systemic treatment. Some patients with a higher risk of local treatments such as thermal ablation or poor efficacy of EBRT also selected BIRS.

The process was performed as recommended in the literature [15]. Briefly, the preoperative plan for each lesion was developed by Seeds Implanting Brachytherapy System (Beijing Astro Technology), based on the CT images. Following the recommendations of the American Brachytherapy Society for prostate cancer and American Association of Physicists in Medicine for the dosimetry of seed implantation [14,15], the prescribed matched peripheral dose was set to an average of 120Gy (range, 100-140Gy). The required amount of 125I seeds and total exposure dose were calculated such that 90% of PTV was exposed to more than 90% of the prescribed dose. A dose-volume histogram was generated, and the seed distribution was adjusted to achieve the optimal level.

125I seeds (Type 6711, Yunke Pharmaceutical) with a diameter of 0.8 mm, length of 4.5 mm, activity of 0.8 or 0.6mCi, and half-life of 59.6 days were selected for all patients. The puncture needles (18 Gauge, Yunke Pharmaceutical) were inserted into the lesions with a distance of 10 to 15mm between each needle under CT guidance, and the seeds were implanted into the lesions. Within 2 hours post-implantation, a CT scan was performed for post-implantation dosimetry assessment. Spiral CT scans were acquired at 5-mm thickness and 5-mm spacing, extending 2cm above and below the most superior and inferior seeds. For tumors exhibiting insufficient radioactivity, timely dose verification is carried out, using the treatment planning system, and then the plan for the insufficient dose area is improved, and seeds are implanted again.

**Single-variable analysis of RSIPR and position of lesions**

The position of lesions was divided into four categories: located in the hepatic dome area, within 2cm from the hepatic portal area, within 2cm from the liver capsule, and located in other areas of the liver. These categories exhibited a priority. The positions of seed implantation had a certain degree of particularity. From a clinical perspective, lesions located in the first three positions may be unsuitable for other local treatments such as thermal ablation and external radiotherapy. For example, the hepatic dome area is usually a blind area for ultrasound, which makes it difficult for ultrasound-guided radiofrequency ablation. Ablation of lesions adjacent to the portal area and capsule carries a higher risk, and the efficacy is affected by the flowing blood. With regard to external radiotherapy, the exposure dose is easily limited due to surrounding tissues, which reduces the efficacy. Accordingly, lesion position was analyzed as an independent factor.

**Tables E**

**Table E1. Conversion and consequence of radioactive seed-induced peritumoral reaction (n=201)**

| Development of RSIPR | No. of lesions with RSIPR (%) |
| --- | --- |
| Conversion | 58 (28.9) |
| Only once | 29 (14.4) |
| Multiple | 29 (14.4) |
| Twice | 20 (9.9) |
| Three times | 6 (3.0) |
| Four times | 3 (1.5) |
| Consequence |  |
| Return to normal tissue* | 82 (40.8) |
| Scarring or atrophy | 26 (12.9) |
| Local progression | 30 (14.9) |

* Defined as the density of RSIPR area in each phase observed to return to the same as that of normal tissue.

**Table E2. Frequency of types of radioactive seed-induced peritumoral reaction**

| Classification | No. of types in conversion (n=38) (%) | No. of final types before returning to normal (n=81) (%) * |
| --- | --- | --- |
| Type I | 3 (7.9) | 54 (66.7) |
| Type II | 14 (36.8) | 14 (17.3) |
| Type III | 8 (21.1) | 8 (9.9) |
| Type IV | 13 (34.2) | 5 (6.2) |

* One lesion could not be classified as type I or II due to the lack of equilibrium phase.

**Table E3. Comparison of serological indicators and** **Child-Pugh classification**

| Indicators and CP class | No. of lesions with RSIPR (%) | | | No. of lesions without RSIPR (%) | | |
| --- | --- | --- | --- | --- | --- | --- |
|  | Before BIRS (n=199) | After BIRS (n=507) | P value* | Before BIRS (n=89) | After BIRS (n=365) | P value |
| ALT | 47 (23.6) | 109 (21.5) | 0.54 | 30 (33.7) | 114 (31.3) ** | 0.66 |
| AST | 27 (13.6) | 99 (19.5) | 0.06 | 34 (38.2) | 164 (45.1) ** | 0.24 |
| TBIL | 13 (6.5) | 50 (9.9) ** | 0.16 | 13 (14.6) | 63 (17.3) ** | 0.54 |
| ALP | 63 (31.7) | 249 (49.1) | <0.001 | 34 (38.2) | 199 (54.67) ** | 0.01 |
| CP class A | 193 (97.5) ** | 482 (95.3) ** | 0.49 | 88 (98.9) | 343 (94.0) | 0.06 |
| CP class B | 5 (2.5) | 23 (4.6) | 0.49 | 1 (1.1) | 22 (6.0) | 0.06 |
| CP class C | 0 (0.0) | 1 (0.2) | 0.49 | 0 (0.0) | 0 (0.0) | 0.06 |

Abbreviations: RSIPR, radioactive seed-induced peritumoral reaction; BIRS, brachytherapy with 125I radioactive seed; ALT, alanine aminotransferase; AST, aspartate aminotransferase; TBIL, total bilirubin; ALP, alkaline phosphatase; CP class, Child-Pugh classification

* Significant difference at α=0.05.

**There was one case of missing or untested data.

**Table E4.** **Univariable analysis for lesions with/without radioactive seed-induced peritumoral reaction (n=290)**

| Variables | No. of lesions with RSIPR (n=201) (%) | No. of lesions without RSIPR (n=89) (%) | P value* |
| --- | --- | --- | --- |
| Sex |  |  |  |
| Male | 143 (71.1) | 75 (84.3) | 0.02 |
| Female | 58 (28.9) | 14 (15.7) | 0.02 |
| Age |  |  |  |
| < 35 | 6 (3.0) | 5 (5.6) | 0.49 |
| 35-60 | 138 (68.7) | 62 (69.7) | 0.49 |
| ≥ 60 | 57 (28.4) | 22 (24.7) | 0.49 |
| Organization source |  |  |  |
| Primary | 75 (37.3) | 51 (57.3) | 0.002 |
| Secondary | 126 (62.7) | 38 (42.7) | 0.002 |
| Histopathological types |  |  |  |
| Hepatocellular carcinoma | 51 (25.4) | 45 (50.6) | <0.001 |
| Other hepatic malignancies: | 150 (74.6) | 44 (49.4) | <0.001 |
| Lesion positions** |  |  |  |
| Hepatic dome | 41 (20.4) | 9 (10.1) | 0.01 |
| Adjacent to the hilar | 38 (18.9) | 24 (27.0) | 0.01 |
| Adjacent to the capsule | 108 (53.7) | 41 (46.1) | 0.01 |
| Other parenchyma | 14 (7.0) | 15 (16.9) | 0.01 |
| Local progression: | 30 (14.9) | 23 (25.8) | 0.03 |
| Hepatocellular carcinoma | 4 (2.0) | 6 (6.7) | - |
| Other hepatic malignancies | 26 (12.9) | 17 (19.1) | - |
| Scarring or atrophy later in the follow-up | 26 (12.9) | 7 (7.9) | 0.21 |

* Significant difference at α=.05.

** Please refer to Appendix E1 for the details of lesion positions.

**Figures E**


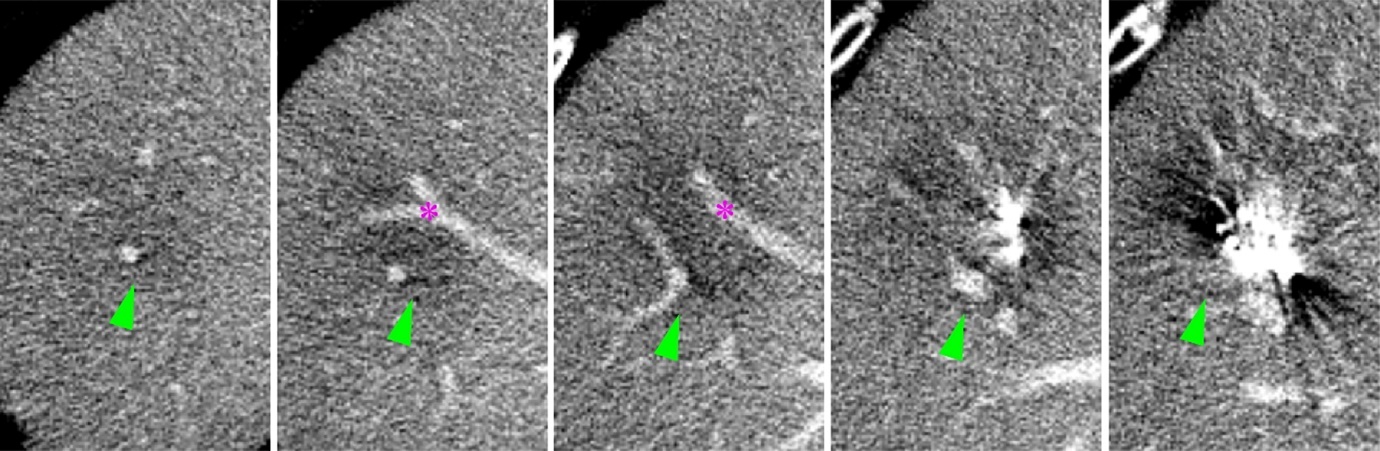


**Figure E1:** A 62-year-old man with hepatocellular carcinoma implanted for 2 months, showing a low-density. Vessels (pink star) in the reaction area (green arrows) were observed without displacement or invasion
